# On the Use of the Whiskers in Feline and Other Animals

**Published:** 1823-05

**Authors:** S. D. Broughton

**Affiliations:** Member of the Royal College of Surgeons; Surgeon to the St. George's and St. James's Dispensary, and to his Majesty's Second Regiment of Life Guards, &c. &c.


**Art. VIII.**—*On the Use of the Whiskers in Feline and other Animals.*

By S. D. Broughton, Member of the Royal College of Surgeons; Surgeon to the St. George's and St. James's Dispensary, and to his Majesty's Second Regiment of Life Guards, &c. &c.

It may not, perhaps, be generally known that, as far as they have been traced, the whiskers which adorn the upper lip in tygers, panthers, leopards, cats, seals, &c. are very copiously supplied with nerves; and altogether in a manner which precludes the possibility of our considering them as merely ornamental appendages.

After macerating the head of a stout cat, and carefully dissecting the face under clear water, I could distinctly trace large branches of nerves pervading the fatty cushions on which the whiskers grow, and sending separately to each bulb or root a filament of the size of a strong thread. These appeared to come from the second branch of the fifth pair, and they were lost in the substance of the bristle. In the Hunterian Museum of the College of Surgeons, a similar development of nerves is exhibited in the seal.

So large and particular a distribution of an exquisitely sensible nerve, it is reasonable to suppose, must be for the purpose of some sensible function. To ascertain this, I put it to the test of experiment, in the following manner:

I placed walls of books upon the floor, so as to resemble the streets of a town opening into each other; and, having closed the eyes of a kitten completely, I set it down to find its way through the lanes of books. It continued to move on wherever a free communication presented itself, holding its head cautiously down close to the floor, and very adroitly avoiding contact with the sides of the walls, the corners of which it also turned without approaching closer than just sufficient to touch the tips of the whiskers slightly, when it always drew back instantaneously. At length it found its way out freely; and I then cut off the whole of the whiskers close to the face, and again set it down, to observe whether this would produce any alteration in its manner. The kitten now showed evident signs of having lost the only remaining means of guiding itself. It struck its head repeatedly against the sides of the walls, run against all the corners, and tumbled over steps placed in its way, instead of avoiding all these as before the removal of the whiskers.

Not the slightest sensation of pain appeared to attend the cutting of the bristles; I should therefore suppose that their substance is insensible, and that it carries on the impressions it receives by vibration, their sensation being propagated through the nerves inserted in the bulbs of the bristles.

In the mouse, I found also a similar development of nerves, proportionably large, in relation to its whiskers.

From these facts, I imagine that certain animals are supplied with whiskers for the purpose of enabling them to stear clear of opposing bodies in the dark. The mammalia, having lips and considerable facial development, are probably furnished with these bristles, or fine tubes of a compact substance, which, whilst they readily yield to pressure, convey the impression and excite the simple sensation of contact; just as the antennæ of the sepia, the lobster, the snail, insects, &c. are to all appearance constructed, for the purposes of the sense of touch.

Animals which seize their prey by night, and such as explore dark passages, are furnished with whiskers. The bat is supposed to avoid walls and houses by the exquisite sensibility of its wings: it seems, however, probable that it is indebted for its safety in this respect to its whiskers, or feelers, as they may be termed.

